# A case report: Delayed gallstone abscess formation 10 years post-cholecystectomy

**DOI:** 10.1016/j.ijscr.2020.11.126

**Published:** 2020-11-30

**Authors:** Erina Quinn, James Capanegro, Joseph Hartigan

**Affiliations:** a Lake Erie College of Osteopathic Medicine, Bradenton, FL, USA; b North Florida Surgeons, Jacksonville, FL, USA

**Keywords:** Cholecystectomy, Gallstones, Abscess, Laparscopic surgery

## Abstract

**Introduction:**

Retained gallstones post-cholecystectomy act as a nidus for abscess formation. It is unusual for intraabdominal abscesses to remain asymptomatic due to its propensity to cause inflammation and irritation to the peritoneum.

**Presentation of case:**

A 73-year-old female presented with acute onset of right-sided abdominal pain and fever. Her past surgical history was significant for a cholecystectomy in 2010, hysterectomy, and partial nephrectomy. She was diagnosed with an intraabdominal abscess secondary to a retained gallstone post-cholecystectomy. She underwent laparoscopic surgery to drain and remove the abscess. The patient's abdominal pain improved, remains afebrile, and is passing stool regularly.

**Discussion:**

Gallbladder perforation is common and is dependent on the integrity of the gallbladder and surrounding structures. It is unusual for an intra-abdominal abscess to develop so late following gallstone spillage. This example brings to light the potential long-term sequelae of gallbladder perforation and future complications.

**Conclusion:**

This case highlights the importance of irrigation of the peritoneal cavity and retrieval any spilled gallstones during surgery in the event of gallbladder perforation.

## Highlights

• Successful removal of gallstones are important to prevent the formation of gallstone abscess and infection.
• An increase of reports of gallstone abscess formation shows the importance of awareness in the surgical community of the occurrence and proper treatment techniques.
• Proper treatment techniques include abscess drainage either surgically or non-invasive and if possible laparoscopic repair of gallstone abscess.

## 1 Introduction

Cholelithiasis affects 20–25 million Americans annually [1]. It is a leading cause for hospital admissions in America with an estimated 1.8 million ambulatory care visits each year [2]. Cholecystectomy is the conventional approach for symptomatic gallstones but can be complicated by gallbladder perforation and gallstone spillage. The incidence of gallstone and bile spillage was approximated to occur in up to 40% of surgeries performed between the years of 1991 and 2015. The most common complication from spilled gallstones is the formation of intra-abdominal abscesses with an incidence of up to 2.9% [3]. We report a rare case of a gallstone abscess that formed 10 years post-cholecystectomy, which is being reported in line with the SCARE criteria [4]. The aim and objective of this study is to increase awareness and prevention of gallstone spillage complications.

## 2 Case description

A 73-year-old female with a past medical history of hypertension and prior cholecystectomy presented to the emergency room with acute onset of abdominal pain of 1-day duration. She described right lower quadrant abdominal pain that was constant and worsening since it started. She reported no prior occurrence. There were no alleviating or exacerbating factors. Her symptoms were preceded by five days of constipation refractory to stool softeners. She was briefly febrile four days prior to admission. Surgical history disclosed laparoscopic cholecystectomy in 2010 complicated with a perforated gallbladder. After being discharged following the surgery, she noted having had recurring fevers for ten days and experienced no other symptoms.

On initial assessment, her exam revealed an elderly woman, who was alert, well-oriented, and in mild distress. She had an oral temperature of 99°F, a heart rate of 89 bpm, blood pressure of 142/63 mmHG, and a respiratory rate of 18 breaths per minute. Her abdominal exam revealed a soft, non-distended stomach with moderate tenderness at the medial right lower quadrant abdomen. There was no guarding or rebound, and she had normal bowel sounds.

**Fig. 1:**
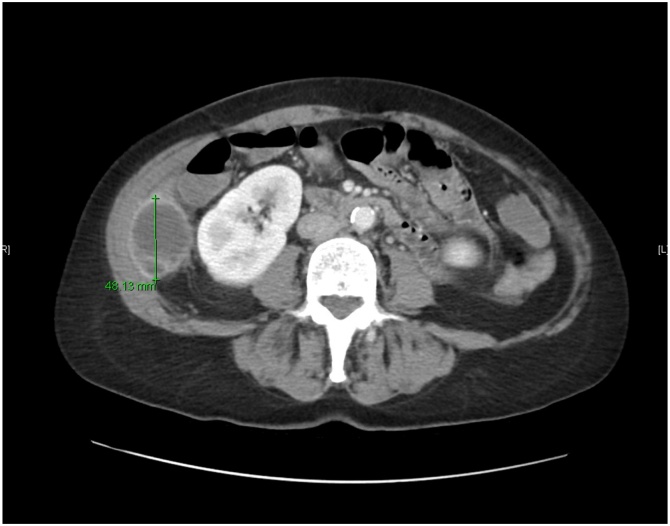
The CT of the abdomen and pelvis with contrast showed a 12.4 × 4.8 × 3.6 cm fluid collection within the right lateral abdominal wall with 2 radiopaque structures resembling gallstones.

**Fig. 2:**
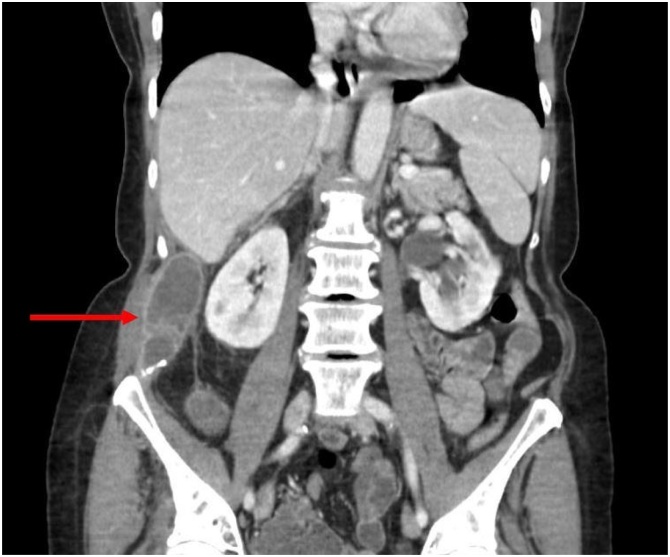
The abscess extended superiorly to the level of Morison's pouch and inferiorly to the right iliac wing.

Initial labs showed an elevated WBC at 15.2 K/mcl, hemoglobin at 13.3 g/dL and a high platelet count of 552 K/mcl. The patient had normal values for the following: sodium at 135 mEq/L, potassium at 3.8 mEq/L, BUN at 16 mg/dL and creatinine at 0.92 mg/dL. A CT of the abdomen and pelvis with contrast showed hepatomegaly along with a 12.4 × 4.8 × 3.6 cm fluid collection within the right lateral abdominal wall with two radiopaque structures resembling gallstones (Fig. 1). The abscess extended superiorly to the level of Morison's pouch and inferiorly to the right iliac wing (Fig. 2).

The patient was admitted and started on intravenous piperacillin-tazobactam. Dr. Joseph Hartigan, who has over 20 years of experience in laparscopic surgery, performed a laparoscopic drainage of the abscess and removal of the gallstones. The origin of the abscess was found near the umbilicus and tracked cephalad up towards the liver. A 15 Blake drain was placed into the abscess cavity and left in place for four days. A phlegmon of approximately 100 cc's of purulent fluid was drained and cultures grew out *E. Coli*. The patient recovered well and was discharged on the 3rd postoperative day. The abscess formed by the gallstones was determined to be the cause of the abdominal pain in this case. The drain was removed during the follow-up appointment two weeks after the surgery. There were no complications. The patient was intrigued in the discovery and thankful for the outcome.

## 3 Discussion

The risk of perforation and spillage of gallbladder contents is high; however, acute complication incidence is low at 0.8%–8.5% [5]. The most common cause of spilled gallstones is through the perforation of the gallbladder from laparoscopic instruments or during gallbladder dissection from the hepatic bed [6,7]. Gallbladders that are surrounded by dense adhesions or acutely inflamed have an increased risk of perforation [8]. In this case, the patient presented ten years post-cholecystectomy with an abscess that grew *Escherichia Coli*. Interestingly, of all the documented cases occurring ten years or more following surgery, there is an overwhelming female predominance of 89%. *E. Coli* is the most common pathogen, as in acute cholecystitis [5]. It is theorized that the presence of nonsterile gallstones in the abdomen caused an inflammatory response leading to the formation of an abscess.

In high-risk situations, it is essential for surgeons to be vigilant for gallbladder perforation and gallstone spillage. If there are spilled gallstones, it is recommended to do preventative measures for infection by irrigation with saline to dilute bile contents and retrieve all stones [9]. Previous studies do not support switching to open surgery as it increases patient morbidity [10]. The recent increase of reported gallstone abscesses illustrates the importance of awareness and technical refinements in the surgical community.

In this case, the surgical management of abscess drainage and stone retrieval agrees with current treatment recommendations [8]. This report emphasizes documentation of spilled gallstones with post-surgical monitoring and prophylaxis to prevent acute or remote abscess formation.

## 4 Conclusion

Laparoscopic cholecystectomy may be complicated by gallbladder perforation and spilled gallstones. These stones can present months to years after the cholecystectomy with infectious complications causing morbidity. Gallstone spillage should be avoided by taking precautions and preventive measures. If spillage occurs, documentation in operative notes, monitoring for complications, prophylaxis, and early and aggressive treatment assure the best outcome.

## Declaration of Competing Interest

None.

## Funding

This research did not receive any specific grant from funding agencies in the public, commercial, or not-for-profit sectors.

## Ethical approval

None.

## Consent

Written informed consent was not obtained from the patient for publication of this case report and accompanying images since there is no are no patient identifying characteristics.

## Author contribution

Erina Quinn – literature review, data analysis, data collection, writing.

James Capanegro – literature review, writing.

Dr. Joseph Hartigan – data collection, data analysis, writing.

## Registration of research studies

Not applicable.

## Guarantor

Erina Quinn.

## Provenance and peer review

Not commissioned, externally peer-reviewed.
